# Histological and histochemical characteristics of the esophagus in local breed donkey (*Equus asinus*)

**DOI:** 10.5455/javar.2023.j647

**Published:** 2023-03-31

**Authors:** Dhyaa A. Abood, Mohammed Sulaiman Dawood, Lamees Ezldeen Mohammed, Abdulkarim Jafar Karim

**Affiliations:** 1 Department of Anatomy & Histology, College of Veterinary Medicine, University of Baghdad, Baghdad, Iraq; 2 Department of Pathology & Poultry Diseases, College of Veterinary Medicine, University of Baghdad, Baghdad, Iraq

**Keywords:** Donkey, histochemical, histological, esophagus

## Abstract

**Objective:**

Certain advantages of donkeys are still not listed as for other equine species. Moreover, donkeys lack comprehensive scientific studies. The present study examines the histological architecture and histochemical characteristics of the esophagus in the Iraqi local breed donkey (*Equus asinus*).

**Materials and Methods:**

Eight esophagus samples were collected from a local breed donkey. Tissue specimens (~1 cm^3^) were collected from the cervical, thoracic, and abdominal regions of the esophagus and processed via routine histological technique. The tissue sections were stained with hematoxylin and eosin, Massons Trichrome, and combined Alcian blue (pH 2.5) plus PAS (AB-PAS).

**Results:**

The esophagus of the local breed donkey had folded mucosa wrapped by thin non-keratinized stratified squamous epithelium. The heights of epithelia of the cervical and thoracic regions of the esophagus were significantly higher than that of the abdominal regions. The lamina propria consisted of dense fibrous tissue that appeared thickest in the thoracic and abdominal regions of the esophagus. The muscularis mucosa disappears at the cervical region, while the thoracic and abdominal regions of the esophagus contain thick, scattered, interrupted bundles of smooth muscle fibers. Tunica submucosa was very thick at the thoracic and abdominal regions of the esophagus, composed of loose connective tissue filled with compound tubular mucoserous esophageal glands. Using a combined AB-PAS stain, mucous alveoli within the esophageal glands indicated strong acidic mucopolysaccharide. Tunica muscularis of the cervical and thoracic regions was built up by striated muscle fibers and turned into smooth type at the abdominal region of the esophagus.

**Conclusion:**

The esophagus of the local breed donkey shows considerable histological similarities with the other mammals that make this species reliable as an experimental model of digestive tissue.

## Introduction

The donkey is a domestic animal in the equine species. It is a herbivores animal from the wild African ass “*Equus africanus*” and has been used as a working animal for at least 5,000 years with many benefits, e.g., donkey’s milk has been used by humans before thousands of years and has become common recently in any country of Europe [1–3]. The esophagus, a hollow organ, is a connecting tube between the pharynx and stomach that allows easy food passing. It consists of tunica mucosa, tunica submucosa, tunica muscularis, and tunica adventitia [4–6]. Previous studies revealed numerous differences in the histological structure according to animal species, such as keratinization of epithelium, the occurrence of the muscularis mucosae, the distribution of serous or mucous esophageal glands, and the cytoarchitecture of the tunica muscularis [7,8]. Muscularis mucosa is absent in dogs [8] and interrupted in local breed dogs [9], whereas the muscularis mucosa is observed in the abdominal region of the esophagus [10]. 

On the other hand, the esophageal glands are located at the first third of the esophagus in pigs [11] and along the entire length of dogs’ esophagus [9]. The unique esophagus character of the donkey is the pigmentation among its different parts, and the common esophageal obstruction is because of a different anatomical entrance to the stomach [12,13]. Among animal species, the cytoarchitecture of the esophagus varies according to types of food and behavior. The morphological and histological structure of the esophagus has been widely described in different animals species, such as one humed camel [5,6], Llama (*Lama glama*) [14], dogs [15] birds, and a wide variety of mammals [16,17] with a profound scarce in the donkey. The present study aimed to inspect the main characteristics of the esophagus in a local breed of donkey.

## Materials and Methods

### Ethical approval

All study methods and preparations were approved (Approval No. PG/1335) by the Animal Care and Use Committee (ACUC) on June 28, 2022 at the Faculty of Veterinary Medicine, Baghdad University, Iraq.

### Preparation of animals

Eight esophagus samples obtained from adult donkeys (3–5 years) were used. Animals were euthanized for a package of research, which includes various organs of the body, to be carried out in the Department of Anatomy & Histology, Faculty of Veterinary Medicine, Baghdad University. The esophagus was removed from the cadaver and washed up with the solution of 0.9% normal saline, followed by immersion in 10% buffered formalin for 48 h. The tissue samples (~1 cm3) of the esophagus were taken from the cervical, thoracic, and abdominal parts of the esophagus, processed by routine histological technique (58°C–60°C), cut at 5–6 μm using a revolving microtome, put on a glass slide and stained with hematoxylin and eosin (H&E), Massons Trichrome (MT), and composite Alcian blue (pH 2.5) plus PAS (AB-PAS) stains [9,18], examined and photographed using Olympus (SC 35) microscope. Histometrical measurements, including the epithelium height, the thickness of the lamina propria, and tunica muscularis were scored and analyzed using the Fiji image analyzer system [19].

### Statistical analyses

Data were expressed as mean ± SE, and one-way analysis of variance was used to detect a significant difference at *p* < 0.05.

## Results

**Figure 1.**
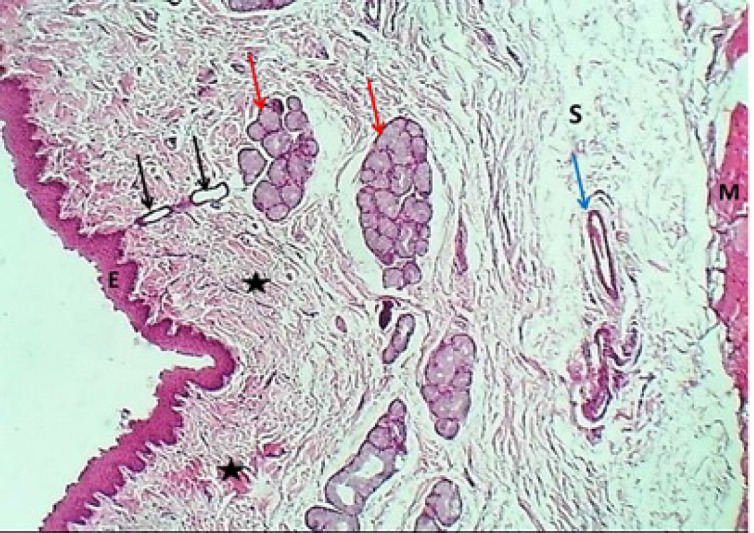
Histomicrograph of esophagus- cervical part shows (E) stratified squamous epithelia, (asterisk) collagenous connective tissue of lamina propria, (black arrows) common excretory duct of esophageal glands, (red arrows) scattered masses of esophageal glands, (S) well vascular tunica submucosa, (M) tunica muscularis contains striated muscles (H&E stain, 40×).

**Figure 2.**
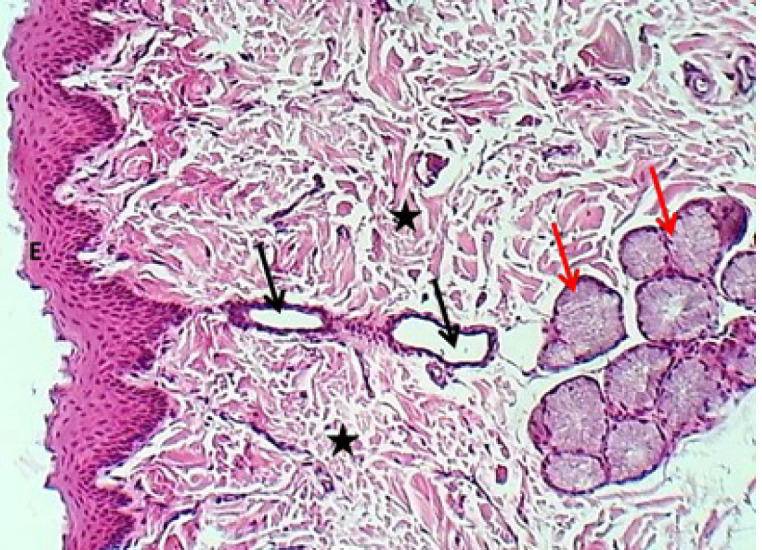
Histomicrograph of esophagus at cervical part shows: (E) thin non-keratinize stratified squamous epithelia, (black arrows) common excretory duct of esophageal glands, tunica submucosa, (M) tunica muscularis contains striated muscles (H&E stain, 40×).

**Figure 3.**
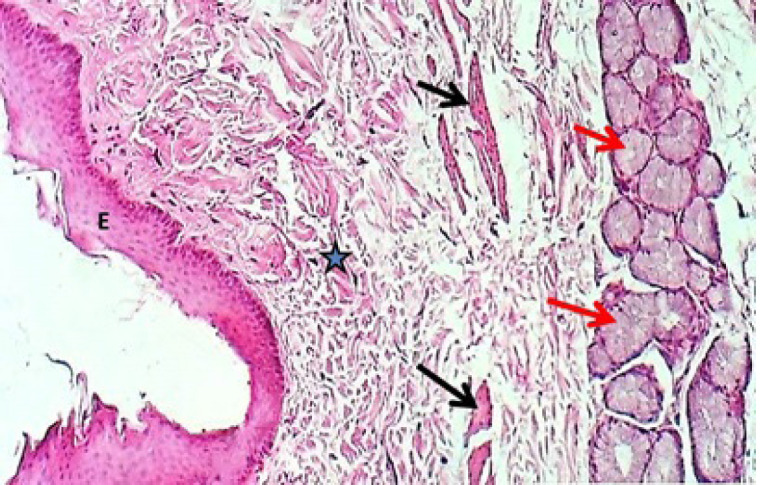
Histomicrograph of esophagus–thoracic part shows: (E) stratified squamous epithelia, (black arrows) interrupted smooth muscle of muscularis mucosa, (asterisk) dense irregular collagenous tissue of lamina propria, (red arrows) thin layer of submucosal esophageal glands (H&E stain, 100×).

**Figure 4.**
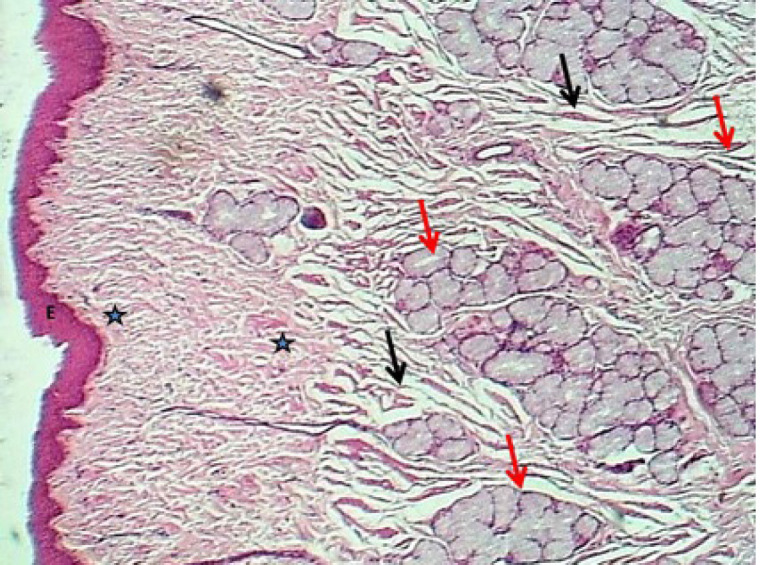
Histomicrograph of esophagus–abdominal part shows: (E) thin non-keratinized stratified squamous epithelia, (asterisk) thick collagenous connective tissue of lamina propria with common excretory ducts, (red arrows) thick layer of submucosal esophageal glands that surrounded by smooth muscle fibers of muscularis mucosa (black arrows) (H&E stain, 40×).

**Figure 5.**
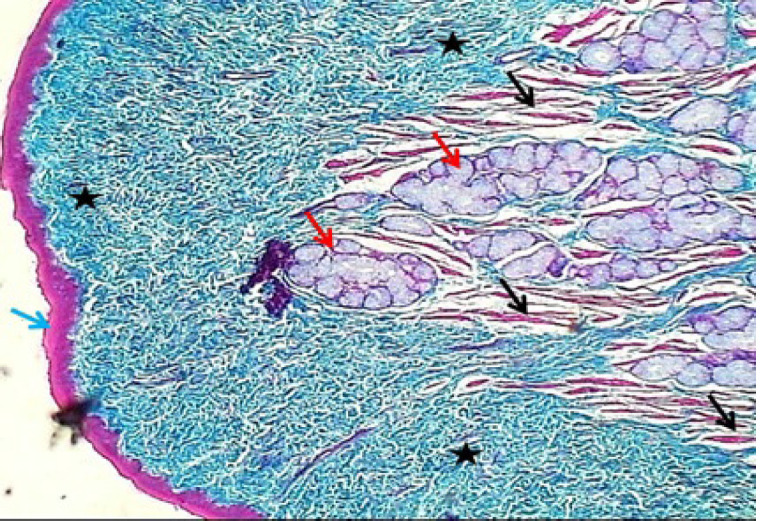
Histomicrograph of the abdominal part of esophagus shows: (blue arrows) thin non-keratinized stratified squamous epithelialis, (asterisk) thick layer of dense irregular collagenous connective tissue of lamina propria with common excretory ducts, (red arrows) thick layer of submucosal esophageal glands that surrounded by smooth muscle fibers of muscularis mucosa (black arrows) (MT stain, 40×).

**Figure 6.**
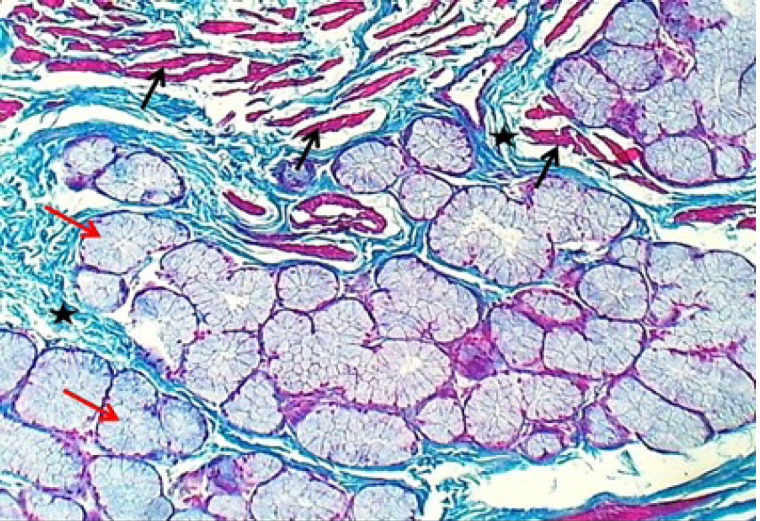
Histomicrograph of esophagus at abdominal part shows (asterisks) dense collagen bundles of lamina propria surrounded esophageal glands, (red arrows) mucous alveoli of esophageal glands, (black arrows) longitudinal bundles of smooth muscle of muscularis mucosa (MT stain, 100×).

Morphologically, the results revealed that the total esophageal length of the local breed donkey ranged from 89–110 cm, 200.32 ± 4.32 gm weight, and 1.54 ± 0.20 cm width. The median thickness of the entire esophageal wall was 980.43 μm. Histologically, esophageal layers consisted of four tunicae; tunica mucosa, tunica submucosa, tunica muscularis, and tunica adventitia. Along the whole parts of the esophagus, huge mucosal folds made by tunica mucosa are covered by thin non-keratinized stratified squamous epithelium with abundant excretory ducts belonging to the esophageal glands (Figs. 1–4). The epithelium was very thin and composed of 7–10 layers of cells. The epithelial heights denoted significant increments in the cervical and thoracic regions (Table 1). Likewise, the thick lamina propria built up by dense irregular collagenous connective tissue denoted significant differences in the thoracic and abdominal regions (Table 1). In the cervical part of the esophagus, both lamina propria and tunica submucosa were merged due to absent muscularis mucosa and build up by dense irregular collagenous connective tissue that showed few esophageal glands (Figs. 1 and 2). On the other hand, the thoracic part of the esophagus showed the lamina propria separated from the submucosa by interrupted bundles of smooth muscle fibers of the muscularis mucosa (Fig. 3). However, this layer consisted of a very thick fibrous connective tissue, heavily occupied by esophageal glands and separated from tunica submucosa by a very thick layer of circular and longitudinal layers of the smooth muscle of muscular mucosa (Figs. 4–6). 

**Figure 7.**
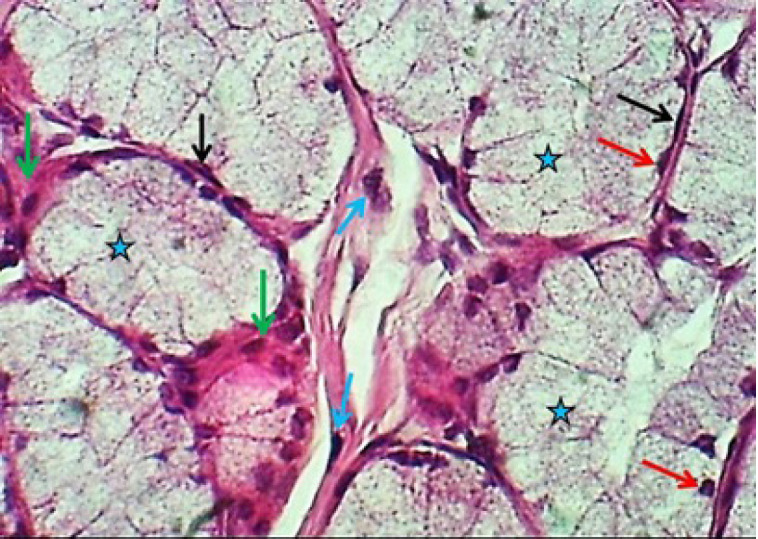
Histomicrograph of compound tubular mucoserous esophageal glands show: (asterisks) alveoli with foamy appearance of mucous granules, (green arrows) little eosinophilic serous demiulons, (black arrows) myoepithelial cells, (red arrows) nuclei of alveolar cells, (blue arrows) inter alveolar fibroblast with collagen fibers (H&E stain, 400×).

**Figure 8.**
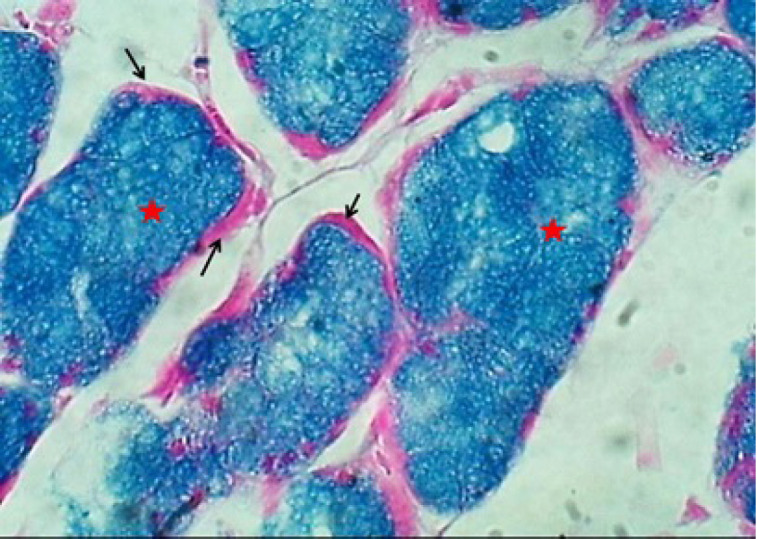
Histomicrograph shows esophageal glands: (asterisks) mucous alveoli contained acidic mucoploysaccharid, (black arrows) denoted thin serous acini secrete neutral zymogen granules (combined AB-PAS stain, 400×).

**Figure 9.**
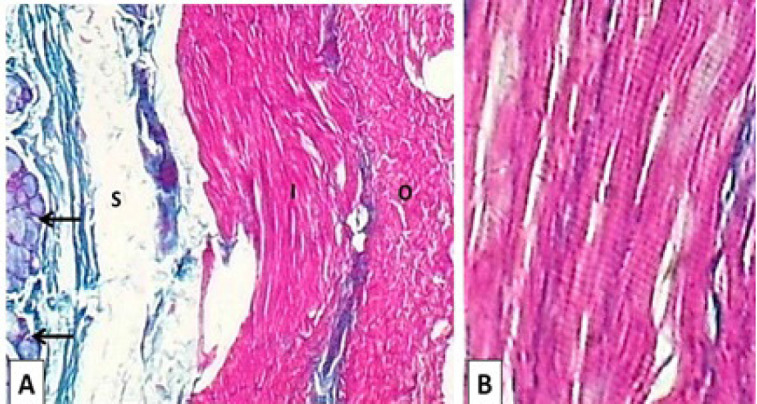
Histomicrograph of esophagus- cervical part shows striated muscle fibers of tunica muscularis (I) inner circular layer and (O) outer longitudinal layer, (S) thin layer of loose connective tissue of submucosa, (arrows) esophageal glands [MT stain; (A) 100×, (B) 400×].

**Figure 10.**
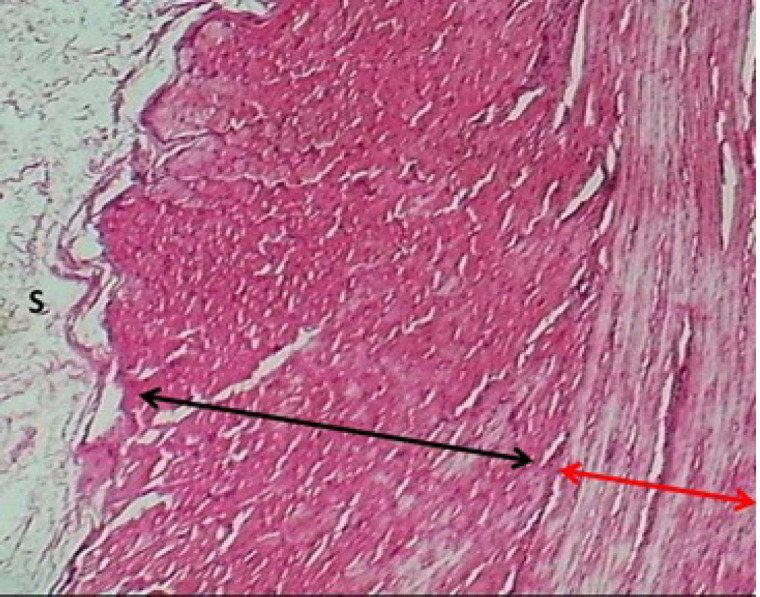
Histomicrograph of abdominal part of esophagus shows tunica muscularis (black double head arrow) thick inner circular layer, (red double head arrow) thin outer longitudinal layer of smooth muscle fibers, (S) submucosa (H&E stain, 40×).

The tunica submucosa was built up by loose connective tissue containing few collagen fibers and adipose tissue occupied by esophageal glands. There were significant increments in the thicknesses of the tunica submucosa of the thoracic and abdominal regions (Table 1). The esophageal glands are composed of tubular mucoserous type and are built up by predominated large mucous alveoli and a few serous secretory units surrounding alveoli (Serous demiulone) (Figs. 6–8). The cervical and thoracic tunica muscularis comprised a thick inner circular layer with an outer longitudinal layer of striated muscle fibers (Fig. 9). In contrast, these inner and outer layers at the abdominal region consisted of smooth muscle fibers (Fig. 10). Loose connective tissue separates the two layers of tunica muscularis from each other. It contains the myenteric plexus, blood vessels, and nerves. The thickness of tunica muscularis at the thoracic and abdominal regions significantly differed (Table 1).

## Discussion

The current study focused on the histological structure of the esophagus of donkeys. Like other large farm animals, the esophagus of the local breed donkey consists of mucosa, submucosa, muscular, and adventia [12,20]. Our finding revealed that the tunica mucosa of the esophagus was lined by non-keratinized stratified squamous epithelium with increased thinning at the abdominal part of the esophagus. The non-keratinized esophageal epithelium was recorded in dogs and humans [17] and guinea pigs [21], while the equine [20], goats [22,23], and guinea pig [24] possess a keratinized type. Our results denoted significant differences in esophageal tunicae thickness along the entire regions of the esophagus. The epithelia were the highest in the cervical region, representing the first upper third of the esophagus that dealt with rough ration. These findings coincided with Dawood et al. [9] in dogs, Devi [17] in humans, Mahmood and Kadhim [25] in Grey Mongooses, and Jones et al. [26] in Agouti, while Kumar et al. [22] recorded that thicker epithelium could be observed in the thoracic region of goat esophagus as compared to the cervical and abdominal parts. The various thicknesses and keratinized epithelia are related to the species‘ natural food for carnivores’ hard diet. This explains that the higher protection of the organ from types of rough and hard foods affects the initial part of the esophagus. The current study revealed that donkeys’ esophagi displayed abundant longitudinally oriented mucosal folds observed in dogs and humans [9,17], in contrast to smooth mucosa with no mucosal folds in one-humped camels [6]. The mucosal folds in feline prevent damage by hard food and allow more dilatation as the food moves down to the stomach [27].

**Table 1.** Epithelial height, thickness of lamina propria, submucosa, and muscularis in cervical, thoracic and abdominal regions of esophagus.

Regions of esophagus	Height (µm) of epitheli	Thickness (µm)		
		Lamina propria	Submucosa	Muscularis
Cervical	91.71 ± 1.37^c^	169.99 ± 4.54^a^	431.27 ± 4.92^a^	449.74 ± 3.76^a^
Thoracic	77.50 ± 2.11^b^	266.97 ± 3.11^b^	456.28 ± 5.39^b^	574.81 ± 4.12^b^
Abdominal	68.21 ± 4.09^a^	361.51 ± 3.11^c^	762.96 ± 4.10^c^	541.34 ± 4.22^c^

Different superscripts refer to significant differences between rows (*p* < 0.05).

The lamina propria of all parts of the esophagus represented a thick layer of fibrous tissue rich with blood and nerve supply [4,6,9]. It was significantly thickest in the abdominal part, while the cervical part contained no muscularis mucosa and scattered bundles in the thoracic part. 

In rabbits, sheep, and goats, the muscularis mucosa contains an interrupted single layer of smooth muscles [28,29], thinly dispersed become thicker in the thoracic region in the Grey Mongoose esophagus [25], and prominent along the entire length of the esophagus of agouti [26] and camel [6]. Our findings revealed that the muscularis mucosa as continuous smooth muscle fibers in the abdominal part coincided with Hussein et al. [6], who referred for continued muscularis mucosae in the camel‘s caudal part of the esophagus. The current study suggests that the muscularis mucosa was clear at the abdominal part of the esophagus and close to the stomach. It has a close relationship with the bulk of the esophageal glands, which helps push the secretions of these glands into the esophageal cavity. The variation in thickness of lamina propria describes its histological structure. The lamina propria formed numerous dermal papillae, which extend into the epithelium and protrude into about 50% of humans’ epithelium [30]. 

Our finding indicated that the compound tubular mucoserous esophageal glands contained numerous mucous alveoli with little serous acinus. These glands were mentioned in one-humped camel by Hussein et al. [6]. The camel esophagus is depicted by marked and massive esophageal glands along its entire length that are purely mucous type, similar to that in dogs [9,15,31]. Vice versa, esophageal glands are not observed in guinea pigs [21,24], goats [22], Grey Mongoose [25], and sheep [32] at any part of the esophagus. The large quantity of mucus secretion protects mucosa against the stomach acid pH. This mucous secretion is a protective barrier for the esophageal mucosa [16]. Our results revealed that with combined AB-PAS stain, the esophageal glands revealed light and dark blue color among serous acini, which refer to strong acidic mucopolysaccharide and weak acidic zymogen granules, respectively. The difference in esophageal gland secretions is attributed to the type and habits of food [9]. Our findings revealed a relation between the increments of tunica submucosa thickness in the thoracic and abdominal regions associated with increases in glandular masses. 

On the other hand, the cervical and thoracic parts of the tunica muscularis of the esophagus were formed by striated muscle fibers, unlike the abdominal part, which was composed of two layers of smooth muscle fibers. This is by the tunica muscularis in the cervical and thoracic parts of the esophagus of carnivores [9,25], sheep, and goats [31]. At the same time, in large ruminants [8,10] and one-humped camels [6], entirely striated muscles. The thoracic and abdominal regions of the esophagus had the thickest tunica muscularis, similar to that recorded in Grey Mongoose [25] and humans [17]. In other animals like agouti, it is formed by an inner circular surrounded by outer longitudinal smooth muscle fibers [25]. In horses, it consists of two layers that appear as obliquely striated muscle, turned into a spiral pattern, or arranged into inner circular and outer longitudinal layers of smooth muscle in the cervical, thoracic, and abdominal parts, respectively. The muscle layer in guinea pigs is formed of the striated type that turns into a smooth type close to the stomach, forming the gastric sphincter [21,24]. At the same time, in goats, it is constructed by a thick inner circular layer surrounded by thinner outer longitudinal striated skeletal muscles [15].

## Conclusion

The esophagus of a local breed donkey (*Equus asinus*) shows considerable histological similarities with the other mammals that make this species fit as an experimental model of digestive tissue.

## List of abbreviations

AB-PAS; combined Alcian blue (2.5pH) plus PAS stain; H&E; Hematoxylin and eosin stain, MT; Massons Trichrome stain,
